# Stress amplifies sex differences in primate prefrontal profiles of gene expression

**DOI:** 10.1186/s13293-017-0157-3

**Published:** 2017-11-02

**Authors:** Alex G. Lee, Megan Hagenauer, Devin Absher, Kathleen E. Morrison, Tracy L. Bale, Richard M. Myers, Stanley J. Watson, Huda Akil, Alan F. Schatzberg, David M. Lyons

**Affiliations:** 1 Department of Psychiatry and Behavioral Sciences, Stanford University, 1201 Welch Rd MSLS Room P104, Stanford, CA 94305-5485 USA; 2 Molecular and Behavioral Neuroscience Institute and Department of Psychiatry, University of Michigan, Ann Arbor, MI USA; 3 HudsonAlpha Institute for Biotechnology, Huntsville, AL USA; 4 Department of Animal Biology, University of Pennsylvania, Philadelphia, PA USA

**Keywords:** Social stress, Sex biased, Prefrontal cortex, Gene expression profiling, Neurotrophins, MAPK signaling, Squirrel monkeys

## Abstract

**Background:**

Stress is a recognized risk factor for mood and anxiety disorders that occur more often in women than men. Prefrontal brain regions mediate stress coping, cognitive control, and emotion. Here, we investigate sex differences and stress effects on prefrontal cortical profiles of gene expression in squirrel monkey adults.

**Methods:**

Dorsolateral, ventrolateral, and ventromedial prefrontal cortical regions from 18 females and 12 males were collected after stress or no-stress treatment conditions. Gene expression profiles were acquired using HumanHT-12v4.0 Expression BeadChip arrays adapted for squirrel monkeys.

**Results:**

Extensive variation between prefrontal cortical regions was discerned in the expression of numerous autosomal and sex chromosome genes. Robust sex differences were also identified across prefrontal cortical regions in the expression of mostly autosomal genes. Genes with increased expression in females compared to males were overrepresented in mitogen-activated protein kinase and neurotrophin signaling pathways. Many fewer genes with increased expression in males compared to females were discerned, and no molecular pathways were identified. Effect sizes for sex differences were greater in stress compared to no-stress conditions for ventromedial and ventrolateral prefrontal cortical regions but not dorsolateral prefrontal cortex.

**Conclusions:**

Stress amplifies sex differences in gene expression profiles for prefrontal cortical regions involved in stress coping and emotion regulation. Results suggest molecular targets for new treatments of stress disorders in human mental health.

**Electronic supplementary material:**

The online version of this article (10.1186/s13293-017-0157-3) contains supplementary material, which is available to authorized users.

## Background

Mood and anxiety disorders occur more often in women than men [1–4]. These mental health disorders are associated with stress-induced modifications in prefrontal cortex [5–7]. Neuroscience research has until recently focused on males [8], and sex differences in stress effects on primate prefrontal profiles of gene expression are not known. Differential gene expression profiles have been reported for diverse brain regions in humans [9, 10] and various monkey species [11, 12], but comparisons across prefrontal cortical regions in primates are uncommon. Prefrontal regions are of interest as elements in brain circuits that mediate stress coping, cognitive control, and emotion [13–16]. Human prefrontal neurobiology is modeled more often in nonhuman primates compared to other animals because of species differences in this region of brain [5].

Sex differences in gene expression profiles were initially thought to be limited in human brain [17]. Recent evidence suggests that many genes may be differentially expressed between the sexes for various brain regions in mice [18–20], monkeys [21], and humans [9, 22, 23]. Certain sex differences may reflect species-specific adaptations [24], but sex differences in stress neurobiology commonly occur in many mammals [25] including squirrel monkeys [26]. Sex differences and stress effects in squirrel monkey prefrontal cortical profiles of gene expression may provide insights on the biology of sex differences in human mental health.

Here, we examine prefrontal cortical gene expression profiles in female and male squirrel monkey adults randomly selected from a larger sample randomized to social separation stress or no-stress treatment conditions. Social separations consistently increase plasma levels of the stress hormone cortisol in female [27] and male [28] squirrel monkeys. Both sexes respond to separations from same-sex conspecifics with prolonged increases in cortisol, but females and males have not been directly compared. In separate studies, females show diminished neuroendocrine stress responses [29] and males show increased hippocampal neurogenesis [30] measured 2–4 weeks after exposure to same-sex social separations. Long-term effects are likewise assessed here for prefrontal cortical profiles of gene expression in both sexes. Gene expression profiling represents discovery research [31] that is not guided by hypotheses about specific genes. Analyses of gene expression profiles demand statistical sophistication [32], but we aim to balance statistics with biological implications in this report.

## Methods

Twenty two male and 48 female squirrel monkey (*Saimiri sciureus*) adults served as subjects. These monkeys were approximately 6–17 years of age determined from birth records or estimated from dentition. All monkeys were housed in climate controlled rooms at ~ 26 °C on 12:12-h light/dark cycles with lights on at 07:00. Cages were cleaned daily and provisioned with fresh drinking water, commercial monkey chow, and a variety of fruit and vegetable supplements. Various toys, swinging perches, and simulated foraging activities were provided for environmental enrichment.

In naturalistic and semi-naturalistic conditions, squirrel monkeys live in sexually segregated groups comprised of multiple females and many fewer males [33]. To simulate this social organization in our control condition, we randomly assigned 10 males to live with a familiar same-sex conspecific in undisturbed pairs and 24 females to live in undisturbed groups each comprised of four familiar same-sex conspecifics. Another age-matched sample of 12 males and 24 females were randomized to the stress treatment condition in which monkeys housed as described above were intermittently removed from same-sex conspecifics at 12–15-week intervals for 6–7 repeated social separations. During each 3-week separation session, monkeys were housed individually in cages that allowed visual, auditory, olfactory, and limited tactile contact between adjacent animals.

### Prefrontal brain tissue collection

Brains from randomly selected monkeys in the stress (*n* = 6 males and 6 females) and no-stress (*n* = 6 males and 12 females) conditions were collected 4 weeks after completion of the treatment conditions when all monkeys were socially housed with same-sex conspecifics. Collection time was selected to assess long-term effects of stress. After euthanasia, brains were collected using established procedures [34] between 08:00 and 10:00 to control for circadian effects. All brains were collected during nonbreeding seasons when circulating sex steroid hormones remain stable at low levels in these seasonally breeding primates [35].

**Fig. 1:**
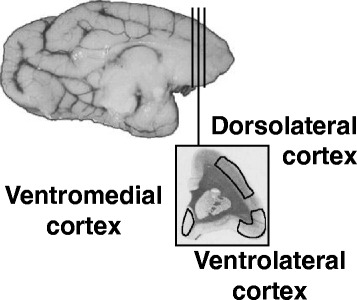
Prefrontal cortical regions. Three regions of interest are depicted on a hemisected coronal tissue section from squirrel monkey brain

Hemisected brains were cut into blocks, flash frozen in isopentane at − 20 °C, and stored at − 80 °C. Fresh frozen blocks from the left brain side immediately anterior to the corpus callosum were cut on a cryostat into 20 μm coronal sections at − 18 °C using RNase-free methods. Serial sections were mounted on glass slides and then stored at – 80 °C. Dorsolateral, ventrolateral, and ventromedial prefrontal cortical regions depicted in Fig. 1 were dissected from coronal sections at 0 °C under a stereo-zoom microscope using RNAse-free instruments and a squirrel monkey brain atlas [36]. All three prefrontal cortical regions were dissected from the same three tissue sections randomly selected from ~ 100 serial tissue sections acquired from each monkey anterior to the corpus callosum on the left brain side. Tissues collected from separate sections were combined into a single sample for each region per monkey.

### BeadChip array hybridization

Tissue samples were homogenized with a motorized pellet pestle, and total RNA was extracted using AllPrep DNA/RNA Micro kits (Qiagen). Samples were amplified (RiboAmp Plus 1.5-round RNA Amplification, Applied Biosystems) for the production of biotinylated cRNA (Bioarray High Yield RNA Labeling, ENZO) and subsequently hybridized to HumanHT-12v4.0 Expression BeadChip arrays (Illumina) that were scanned on a BeadStation system following manufacturer’s instructions. A total of 90 BeadChip arrays were used to generate gene expression profiles for dorsolateral, ventrolateral, and ventromedial prefrontal cortex from 18 females and 12 males. No pooling of samples across monkeys or regions was necessary. Prefrontal cortical regions, sex, and stress treatment conditions were randomly counterbalanced across separate BeadChip arrays.

### qPCR validation

To verify array results, we focused on *HSD11B1* and *CACNG2* expression in each of the three prefrontal cortical regions assessed by quantitative real-time polymerase chain reaction (qPCR) with *ACTB* as the reference gene. Primers designed with Primer-BLAST (NCBI) for Sybr Green assays with parameters set as described elsewhere [37] are as follows: *HSD11B1* forward 5′ ATGTGGTGGTGACAGCGAG 3′; *HSD11B1* reverse 5′ TATAGTGCGCTGAGGCTGCT 3′; *CACNG2* forward 5′ GGCCCTGTCCTTCATCATC 3′; *CACNG2* reverse 5′ GCAGAGGCCTGGAGGTAGT 3′; *ACTB* forward 5′ CAAGGCCAATCGTGAGAAGA 3′; and *ACTB* reverse 5′ AGAGGCGTACAAGGAAAGCA 3′. The same samples analyzed by array were used for qPCR reactions conducted following manufacturer’s instructions (Biorad, SsoAdvanced Universal SYBR Green) under these conditions: 250 nM primers, 50 mM Na^+^, 3 mM Mg^++^, and 1.2 mM dNTP, using the MxPro3000 (Stratagene). Results from qPCR were analyzed as described elsewhere [38].

### Array data processing

Each BeadChip array has more than 47,000 probes designed for the human genome. To identify probes suited for squirrel monkeys, we compared probe sequences against the squirrel monkey genome (Broad Institute, GCA_000235385.1) using BLAT [39]. Selected probes were required to match a single continuous segment of the squirrel monkey genome at 95% homology or greater. For the 11,209 selected probes, expression values were imported into R [40], background corrected using maximum likelihood estimation [41], log2 transformed, and quantile normalized. Normalized data were analyzed for outliers using box plots, sample-sample correlation matrices, and principle components analysis. Two males from the stress condition were excluded as outliers with a median sample-sample correlation of 0.946 compared to 0.980 for all remaining samples. Probes not associated with known genes were discarded. Using these procedures, we analyzed 8853 probes interrogating 7299 unique genes in 18 females and 10 males for 3 prefrontal cortical regions.

### Statistical analysis

A multi-level regression model also known as a hierarchical linear model or a mixed-effects model [42] was used to assess stress, sex, and prefrontal cortical region main effects in expression data for each probe. These analyses were performed in R (R v.3.3.0: https://cran.r-project.org, RStudio v.0.99.896: http://www.rstudio.com/) using the *lme()* function in the *nlme* package (https://cran.r-project.org/package=nlme) with a default correlation structure (no within-group correlation) and method (REML: maximizing the restricted log-likelihood). Our model included the fixed effects of stress (reference level = control condition), sex (reference level = female), and prefrontal region (reference level = dorsolateral prefrontal cortex) with repeated measures accounted for by including individual subjects as a random effects variable (random intercept). Age was unevenly distributed across stress and sex groupings, and in humans, age alters frontal cortical gene expression profiles [43]. We therefore included age class as a fixed effect covariate in our model for squirrel monkeys (younger adult = − 1, middle-age adult = 0, older adult = 1). Age class categories were used to accommodate age estimates based on dentition. All test statistics were evaluated with two-tailed probabilities, and Benjamini-Hochberg false discovery rate corrected *q* values were computed for each nominal *P* value to address the multiple comparisons issue [44].

To further explore regional differences in gene expression profiles, a single summary statistic for regional effects on expression of each probe was extracted using a likelihood ratio approach. Specifically, we used *anova()* function in R to compare the full multilevel model described above with a model that lacked the fixed effect term for region [45]. For probes with significant regional effects (*P* < 0.05) that survived multiple testing correction (*q* < 0.05), hierarchical cluster analysis with average linkage was used to assess relations between regions in SYSTAT13 (http://systatsoftware.com/).

We also examined how regional differences in gene expression profiles of prefrontal cortex in squirrel monkeys compare to humans. To identify regions of human prefrontal cortex with gene expression profiles similar to regions of squirrel monkey prefrontal cortex, we compared monkey results to Allen Brain Atlas human brain microarray data (http://human.brain-map.org, downloaded 12/2015). Allen Brain Atlas microarray data span 160 cortical and subcortical brain regions in high-quality post-mortem tissue from healthy adult men and women [46]. Preprocessed microarray data from the Allen Brain Atlas website were downloaded as *z*-scores with log2 expression data for each probe transformed so that the mean for the entire brain was equal to 0 and the standard deviation equal to 1. For our purposes, we then extracted human expression data from 20 specific regions of frontal or anterior cingulate cortex listed in Additional file 1: Table S1 and re-centered and rescaled these data for each probe. We also centered and scaled our squirrel monkey data for each probe to make similar formats for monkeys and humans, and averaged data for each probe by region. Average regional gene expression signatures for both species were then compared using a region-region correlation matrix and hierarchical clustering.

To identify molecular pathways in monkey gene expression data, probes with significant sex differences (*P* < 0.05) that survived multiple testing correction (*q* < 0.05) were uploaded to DAVID6.7 [47] for annotation with Kyoto Encyclopedia of Genes and Genomes (KEGG) terms. The complete set of 8853 probes that targeted 7299 unique genes identified as suitable for squirrel monkeys served as background for all pathway analyses. Chromosome locations are unknown for many squirrel monkey genes, and therefore we used a comparative approach with Ensembl BioMart to identify genes on sex chromosomes in humans and marmoset monkeys (*Callithrix jacchus*, C_jacchus3.2.1). Like squirrel monkeys, marmosets are also New World primates. Effect sizes for sex differences in gene expression were calculated using Eta squared *η* ^2^ [48] and assessed by analysis of variance in SYSTAT13 with stress as a between-subjects factor and region considered a within-subjects factor.

## Results

**Fig. 2:**
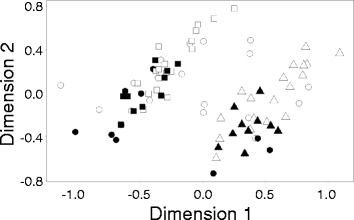
Two primary gradients identified by multi-dimensional scaling of array data. Dimensions 1 and 2 reflect differences between the sexes and variation between prefrontal cortical regions. Females (*white shapes*) are primarily in the upper right with males (*black shapes*) in the *lower left*. Ventrolateral prefrontal cortex (*triangles*) is primarily in the lower right with dorsolateral (*circles*) and ventromedial (*squares*) prefrontal cortical regions in the *upper left*

Age class effects did not survive multiple testing correction for any of the 8853 probes that targeted 7299 unique genes. None of the probes likewise survived multiple testing correction for interactions between stress, sex, and prefrontal cortical regions in expanded models. Therefore, we focus on main effects illuminated by two primary gradients in the data identified by multi-dimensional scaling (Fig. 2).

### Regional effects

Prefrontal cortical region main effects were discerned for 5884 probes that targeted 5155 unique genes (2.6 × 10^−33^ ≤ *P* ≤ 0.05). Nominal *P* values for 5557 of these probes that targeted 4905 genes survived multiple testing correction (2.3 × 10^−29^ ≤ *q* ≤ 0.05; Additional file 2: Table S2). More than 240 probes with region main effects (*P* < 0.05) that survived multiple testing correction (*q* < 0.05) targeted X chromosome genes in humans and marmoset monkeys (Additional file 2: Table S2). None of the probes in our gene expression profiling analyses of squirrel monkeys targeted Y chromosome genes in humans or marmosets.

**Fig. 3:**
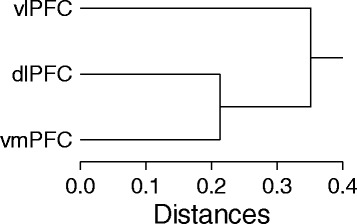
Relations between regions. Hierarchical cluster analysis of 5557 probes with prefrontal cortical region main effects (*P* < 0.05) that survived multiple testing correction (*q* < 0.05). Mean expression per probe for each region is used to identify relations between regions. Abbreviations: vlPFC ventrolateral prefrontal cortex, dlPFC dorsolateral prefrontal cortex, vmPFC ventromedial prefrontal cortex

Hierarchical cluster analysis of probes with prefrontal region main effects (*P* < 0.05) that survived multiple testing correction (*q* < 0.05) revealed that ventromedial and dorsolateral regions were more similar to one another compared to ventrolateral prefrontal cortex (Fig. 3). The number of probes differentiating prefrontal cortical regions in within-subjects pairwise comparisons supported the conclusion that ventromedial and dorsolateral regions resembled one another more than ventrolateral prefrontal cortex. Ventromedial and dorsolateral regions differed on 1302 probes (*P* < 0.05, *q* < 0.05). Using the same pairwise approach, more than three times as many probes differed between ventrolateral prefrontal cortex compared to either ventromedial (4879 probes) or dorsolateral (4346 probes) prefrontal cortical regions (*P* < 0.05, *q* < 0.05).

Cross-species comparisons indicated that squirrel monkey dorsolateral prefrontal cortex resembled middle frontal gyrus (*R* = 0.197, *P* = 4.98 × 10^−87^) and precentral gyrus (*R* = 0.183, *P* = 1.06 × 10^−74^) in humans portrayed in Allen Brain Atlas microarray data. Squirrel monkey ventromedial prefrontal cortex resembled parolfactory (or subcallosal) gyri (*R* = 0.111, *P* = 1.71 × 10^−28^) and gyrus rectus (*R* = 0.107, *P* = 2.00 × 10^−26^) in humans. Squirrel monkey ventrolateral prefrontal cortex resembled posterior orbital gyrus (*R* = 0.047, *P* = 2.49 × 10^−6^) and frontal pole (*R* = 0.063, *P* = 2.49 × 10^−6^) in humans.

### Sex effects

Sex difference main effects in squirrel monkeys were discerned for 1270 probes that targeted 1219 unique genes (8.5 × 10^−8^ < *P* < 0.05). Nominal *P* values for 82 of these probes that targeted 80 unique genes survived multiple testing correction (3.8 × 10^−4^ ≤ *q* ≤ 0.05; Additional file 3: Table S3). Of these 82 probes, 19 had nominally significant age class effects (*P* < 0.05) that did not survive multiple testing correction (Additional file 3: Table S3). For these 19 probes, unbalanced age distributions could potentially contribute to differences between the sexes but this possibility does not likely explain sex differences for the remaining 63 probes.

Most genes expressed differently between the sexes across squirrel monkey prefrontal cortical regions did not reside on sex chromosomes. None of the probes targeted Y chromosome genes and only 2 of 82 probes with sex difference main effects (*P* < 0.05) that survived multiple testing correction (*q* < 0.05) targeted X chromosome genes in humans and marmoset monkeys. Both of these genes (*SLC25A5* and *TSC22D3*) were expressed at higher levels in squirrel monkey females compared to males across all three prefrontal cortical regions.

Many probes expressed at higher levels in squirrel monkey females compared to males targeted autosomal genes. For example, *HSD11B1* is located on chromosome 1 in humans and this gene was expressed at higher levels in squirrel monkey females compared to males across prefrontal cortical regions discerned by two probes on the array (Additional file 3: Table S3). Array results for both probes correlated with *HSD11B1* expression determined by qPCR (Additional file 4: Table S4) and qPCR determinations were greater in females compared to males (*F*(1,24) = 17.2, *P* < 0.001 for the sex main effect). A region main effect was also discerned by qPCR (*F*(2,48) = 21.3, *P* < 0.001) and by both array probes (Additional file 2: Table S2) for *HSD11B1* expression*.* Regional differences do not likely explain correlations because qPCR correlated with array measures for *HSD11B1* regardless of whether prefrontal cortical regions were analyzed separately or combined (Additional file 4: Table S4). Comparable correlations were also identified for another autosomal gene (*CACNG2* in Additional file 4: Table S4) that differed significantly between prefrontal cortical regions determined by array (Additional file 2: Table S2) and by qPCR (*F*(2,48) = 6.8, *P* = 0.002 for the region main effect).

Of the 82 probes with sex difference main effects (*P* < 0.05) that survived multiple testing correction (*q* < 0.05), 64 (78%) were increased across prefrontal cortical regions in squirrel monkey females compared to males. Conversely, 18 of 82 probes (22%) were increased across prefrontal cortical regions in males compared to females. Molecular pathways of genes targeted by probes with sex difference main effects were assessed using DAVID6.7 [47].

For genes with increased expression in females compared to males, two KEGG pathway terms had nominal *P* values that survived multiple testing correction (hsa04722 neurotrophin signaling pathway, 7.7-fold enrichment, *P* = 6.0 × 10^−4^, *q* = 0.017; and hsa04010 MAPK signaling pathway, 5.5-fold enrichment, *P* = 2.1 × 10^−4^, *q* = 0.012). More than half of the genes identified in these pathways were classified under both neurotrophin and MAPK signaling (Table 1). No KEGG terms survived multiple testing correction for genes with increased expression in males compared to females.

**Table 1:** Genes in two molecular pathways. Specific genes overrepresented in neurotrophin and MAPK signaling pathways with greater expression in squirrel monkey females compared to males (mean ± SEM) across prefrontal cortical regions

KEGG pathway	Gene	Females	Males	*P*	*q*
Neurotrophin signaling	*CAMK2G*	7.63 ± 0.08	7.16 ± .012	0.00001	0.009
MAPK signaling	*TGFBR2*	8.77 ± 0.08	8.24 ± .011	0.00044	0.048
	*STK4*	7.94 ± 0.07	7.38 ± 0.10	0.00006	0.016
	*MAP3K4*	8.31 ± 0.08	7.87 ± 0.12	0.00001	0.009
Neurotrophin and MAPK signaling	*TRAF6*	6.54 ± 0.06	6.19 ± 0.09	0.00006	0.016
	*CRKL*	11.88 ± 0.05	11.58 ± 0.06	0.00010	0.020
	*RPS6KA2*	11.85 ± 0.05	11.55 ± 0.06	0.00009	0.020
	*MAP2K5*	5.93 ± 0.06	5.72 ± 0.07	0.00029	0.038
	*MAPK10*	12.09 ± 0.04	11.82 ± 0.05	0.00002	0.012

To test for greater variation in gene expression associated with estrous cycle-related hormonal fluctuations, we followed a statistical approach described elsewhere by other investigators [49]. Coefficients of variation (CV) were calculated separately for no-stress control females and control males for each of the 82 probes with sex difference main effects (*P* < 0.05) that survived multiple testing correction (*q* < 0.05). Twelve females from the control condition were examined to increase chances of assessing different stages of the estrous cycle. Sex-specific CVs were not significantly greater in females compared to males for any prefrontal cortical region.

### Stress effects

**Fig. 4:**
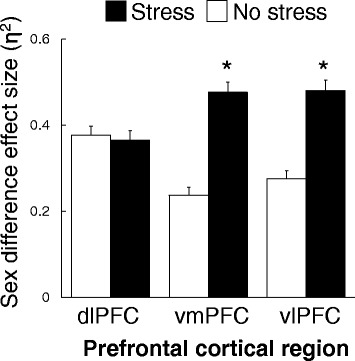
Certain sex differences are amplified by stress. Effect sizes (*η* ^2^) for sex differences are presented in stress and no-stress conditions for 82 probes that targeted 80 unique genes expressed in prefrontal cortical regions (mean ± SEM). The stress-by-region interaction described in the text reflects stress induced disparities in sex difference effect sizes for ventromedial and ventrolateral regions (*P < 0.001) but not dorsolateral prefrontal cortex. Abbreviations: dlPFC dorsolateral prefrontal cortex, vmPFC ventromedial prefrontal cortex, vlPFC ventrolateral prefrontal cortex

None of the stress main effects nor interactions survived multiple testing correction. Nevertheless, effect sizes for sex differences were greater in stress compared to no-stress conditions (Fig. 4) as discerned by a stress main effect (*F*(1,162) = 46.3, *P* = 1.9 × 10^−10^) and a stress-by-region interaction (*F*(2,324) = 26.5 *P* = 2.1 × 10^−11^). Effect sizes for sex differences were greater in stress versus no-stress conditions for ventromedial (*F*(1,162) = 63.1, *P* = 2.4 × 10^−11^) and ventrolateral (*F*(1,162) = 43.2, *P* = 6.5 × 10^−11^) prefrontal cortical regions but not dorsolateral prefrontal cortex (*P* = 0.687). Three of 82 probes with sex difference main effects (*P* < 0.05) that survived multiple testing correction (*q* < 0.05) showed a nominally significant stress main effect (*P* < 0.05) but none of the stress effects survived multiple testing correction.

The top 25 genes with the largest disparity in effect sizes for sex differences in stress versus no-stress conditions in ventromedial and ventrolateral prefrontal cortical regions are depicted in Table 2. Three of these genes (*MAP3K4*, *TRAF6*, *CRKL*) were identified earlier in MAPK and neurotrophin signaling pathways (Table 1). Another three genes (*SLC6A1*, *SPRY4*, *TSC22D3*) with large disparities in effect sizes for sex differences in stress versus no-stress conditions are discussed below. Five of the 25 genes in Table 2 (*MAP3K4*, *KCNIP1*, *HHIP*, *SLC24A2*, *TRIM4*) had nominally significant age class effects (*P* < 0.05) that did not survive multiple testing correction (Additional file 3: Table S3). For these 5 genes, unbalanced age distributions could potentially contribute to stress related disparities in effect sizes for sex differences but this possibility is not likely for the remaining 20 genes (80%) depicted in Table 2.

**Table 2:** Top 25 genes with the largest disparity in effect sizes for sex differences in stress versus no-stress conditions. Effect sizes (*η* ^2^) for sex differences in stress and no-stress conditions are presented for ventrolateral (vlPFC) and ventromedial (vmPFC) prefrontal cortical regions. F > M signifies genes with greater expression in females compared to males, and M > F signifies the reverse

Gene	Biological function	vlPFC		vmPFC		Effect direction
		Stress	No-stress	Stress	No-stress	
*MAP3K4*	MAPK signaling	0.65	0.11	0.57	0.05	F < M
*CRKL*		0.66	0.13	0.59	0.16	F < M
*TRAF6*		0.30	0.18	0.73	0.11	F < M
*SLC6A1*	Transporter activity	0.69	0.33	0.80	0.26	F < M
*SLC24A2*		0.30	0.08	0.63	0.12	F < M
*SLC44A1*		0.71	0.30	0.30	<0.01	F < M
*KCNIP1*		0.40	0.07	0.75	0.08	F < M
*TSC22D3*		0.56	0.15	0.58	0.07	F < M
*HHIP*	Brain development	0.57	0.10	0.39	0.02	F < M
*SPRY4*		0.80	0.33	0.47	0.06	F < M
*MEIS1*		0.47	<0.01	0.72	0.07	F < M
*ARCN1*		0.56	0.31	0.76	0.18	F < M
*DET1*	Protein assembly	0.78	0.04	0.49	0.01	F< M
*RBBP5*		0.38	0.02	0.70	0.03	F < M
*TRIM4*		0.71	0.14	0.46	0.32	F < M
*ZDHHC6*	Catalytic activity	0.84	0.26	0.83	0.44	F < M
*ABCF2*		0.51	0.05	0.52	0.10	F < M
*HELQ*		0.60	0.18	0.69	0.35	F < M
*WDR33*	RNA processing	0.69	0.16	0.66	0.21	F < M
*RBMS1*		0.59	0.34	0.78	0.05	F < M
*CLDND2*	Miscellaneous	0.72	0.16	0.80	0.09	M > F
*LRCH1*		0.84	0.16	0.85	0.31	F < M
*TM9SF1*		0.69	0.11	0.57	0.09	F < M
*ANKRD37*		0.69	0.05	0.62	0.23	F < M
*CAP1*		0.54	0.28	0.41	0.10	F < M

## Discussion

We examined gene expression profiles for dorsolateral, ventrolateral, and ventromedial prefrontal cortical regions from female and male squirrel monkey adults randomly selected from a larger sample randomized to stress or no-stress conditions. Human-specific probes were aligned to the monkey genome and those with ≥ 95% sequence homology were selected for analysis. Results indicate extensive variation between prefrontal cortical regions in the expression of numerous autosomal and sex chromosome genes. Robust sex differences were also discerned across prefrontal cortical regions in the expression of mostly autosomal genes. Genes with increased expression in females compared to males were overrepresented in MAPK and neurotrophin signaling pathways. Many fewer genes with increased expression in males compared to females were discerned, and no molecular pathways were identified. Effect sizes for sex differences were greater in stress compared to no-stress conditions for ventromedial and ventrolateral prefrontal cortical regions but not dorsolateral prefrontal cortex.

Differential gene expression profiles have been reported for diverse brain regions in humans [9, 10] and various monkey species [11, 12] but comparisons between primate prefrontal cortical regions are uncommon. We found that gene expression profiles for prefrontal cortical regions in squirrel monkeys resembled profiles of anatomically comparable prefrontal regions in humans. Squirrel monkey dorsolateral and ventromedial gene expression profiles more closely resembled one another compared to ventrolateral prefrontal cortex. Dorsolateral prefrontal cortex is involved in working memory and cognitive control whereas ventral regions play a role in emotion regulation [50–52]. How prefrontal cortical gene expression profiles mediate behavioral functions remain to be explored but primate prefrontal profiles are spatially distinct.

Robust sex differences were identified across prefrontal cortical regions despite initial suggestions that sex differences in gene expression profiles are limited in human brain [17]. Recent results instead suggest that many genes are differentially expressed between the sexes for various brain regions in mice [18, 19], monkeys [21], and humans [9, 22, 23]. Our results agree and confirm that 82 probes that targeted 80 unique genes are differentially expressed in male and female squirrel monkeys across prefrontal cortical regions.

Similar sex differences in different species raise the possibility of a conserved sexual signature in gene expression profiles of brain. Reinius et al. [21] found 85 genes differentially expressed between the sexes for occipital cortex in humans and cynomologus macaque monkeys. Only two of these genes were differentially expressed in occipital cortex of female and male marmoset monkeys [21]. These two genes were not examined in our study of squirrel monkeys but another gene in squirrel monkeys overlapped with the Reinius et al. [21] list for humans and macaques. In all three species, *SLC6A1* is upregulated in cerebral cortex of females compared to males. Molecular genetic phylogenies suggest that macaques more closely resemble humans than do either squirrel monkeys or marmosets [53] but macaques and squirrel monkeys are polygamous and sexually dimorphic in size whereas marmoset males and females are monogamous and similar in size [54].

*SLC6A1* encodes gamma-aminobutyric acid (GABA) transporter 1 (GAT-1) which terminates GABA neurotransmission via synaptic reuptake specifically in cerebral cortex [55]. Selective GAT-1 inhibitors such as tiagabine have anxiolytic and antidepressant effects in mice [56], and novel GAT-1 inhibitors are now being tested as potential treatments for anxiety and depression [57]. Studies of GAT-1 inhibitors have thus far focused primarily on males [56, 57], but our results suggest that sex differences warrant attention.

Another autosomal gene expressed at higher levels in squirrel monkey females compared to males across prefrontal cortical regions is *HSD11B1.* This gene encodes an enzyme that regulates glucocorticoid exposure by catalyzing conversion of inactive to active glucocorticoids [58]. A recent study of 134 healthy humans between the ages of 20 and 81 years reported increased skeletal muscle *HSD11B1* expression that correlated with age in women but not men [59]. We did not find significant correlations between age and *HSD11B1* expression in any prefrontal cortical region for our smaller sample of female or male squirrel monkeys.

In humans with major depression, we recently identified *FGF9* as a gene of interest [60]. Administration of *FGF9* to male rats increases anxiety and depression-like behavior [60], and *FGF9* stimulates expression of *SPRY4* in vitro [61]. In squirrel monkeys, *FGF9* did not differ between the sexes but *SPRY4* was increased across prefrontal cortical regions in females compared to males. *SPRY4* encodes a negative regulator that suppresses neurotrophic functions [62, 63]. Inhibition of negative regulators that suppress neurotrophic functions may provide a novel approach for the development of new antidepressant medications [60].

Differential expression of single genes is noteworthy, but collective differences in multiple genes along functional pathways provide further insights [64]. Both MAPK and neurotrophin signaling pathways were significantly enriched with genes expressed at higher levels in squirrel monkey females compared to males across prefrontal cortical regions. More than half of the genes enriched in these pathways were associated with both MAPK and neurotrophin signaling. Neurotrophins activate MAPK signaling via extracellular signal-regulated kinases, and MAPK signaling is also activated by cJun N-terminal kinases elicited by cellular stress and pro-inflammatory conditions [65]. These findings agree with evidence that women may be more vulnerable to the depressogenic effects of inflammation compared to men [66].

In human dorsal prefrontal cortex, the majority of genes differentially expressed between the sexes are autosomal and not sex chromosome genes [23]. Most sex differences in squirrel monkey prefrontal cortical regions are likewise autosomal, but two X chromosome genes were identified. Both *SLC25A5* and *TSC22D3* may have escaped X chromosome inactivation [67, 68] insofar as higher expression levels were discerned in squirrel monkey females compared to males across prefrontal cortical regions. *TSC22D3* is induced by stress levels of glucocorticoids [69] and encodes a leucine zipper protein linked to MAPK signaling [70] and neuroplasticity in depression [71]. *SLC25A5* encodes a mitochondrial protein [72] associated with anxiety in mice [73].

None of the stress main effects nor interactions in our study survived multiple testing correction possibly because of exposure to a mild intermittent social stressor. Nevertheless, effect sizes for sex differences were greater in stress compared to no-stress conditions for ventromedial and ventrolateral prefrontal cortical regions but not dorsolateral prefrontal cortex. Functional differences between regions suggest that stress modulates effect sizes for sex differences in prefrontal cortical regions involved in emotion regulation and not working memory nor cognitive control [50–52]. Two genes provide examples. Effect sizes for sex differences in *SLC6A1* and *SPRY4* expression were two to sevenfold greater in stress compared to no-stress conditions for ventromedial and ventrolateral prefrontal cortical regions. Increased *SLC6A1* and *SPRY4* expression in females compared to males during stressful conditions may respectively accelerate termination of GABA neurotransmission and inhibit neurotrophic functions. As discussed above, GABA neurotransmission and neurotrophic functions have been both implicated in mood and anxiety disorders [56, 60].

Greater effect sizes for sex differences in stress compared to no-stress conditions have additional implications for calls to include both sexes in preclinical animal research [74]. Studies not explicitly focused on stress often include manipulations or conditions that are inherently stressful. Our findings suggest that certain sex differences are amplified in stressful situations. If our findings from monkeys hold true for humans and other animals, then studies that include unintentional stressors with males alone may overlook significant sources of biological variability.

Our results should be interpreted in the context of potential limitations. Objectively similar stressors may be viewed differently by the sexes and small samples restricted our capacity to detect stress, sex, and region interactions. Although we analyzed 8853 probes that targeted 7299 unique genes, many probes designed for humans do not match the squirrel monkey genome and were not considered. Our array results require further validation, and bulk tissue samples do not necessarily reflect specific cell types or cellular stress states. Tissue samples were collected during non-breeding seasons when circulating sex steroid hormones remain stable at low levels, but organizational effects of sex steroids on brain development were not considered.

## Conclusions

Prefrontal cortical regions in squirrel monkey adults differed in the expression of numerous autosomal and sex chromosome genes. Robust sex differences were also identified across prefrontal cortical regions in the expression of mostly autosomal genes. Stress amplified sex difference effect sizes for gene expression profiles in prefrontal cortical regions involved in stress coping and emotion regulation. Results suggest new molecular targets for sex-specific treatments of stress disorders in human mental health.

## Additional files

Additional file 1: Table S1. Human brain regions of interest. Specific regions of human frontal cortex and anterior cingulate cortex in Allen Brain Atlas array data (http://human.brain-map.org) that we compared with squirrel monkeys. (XLSX 11 kb)

Additional file 2: Table S2. Prefrontal cortical region effects. Genes targeted by probes with prefrontal cortical region main effects (*P* < 0.05) that survived multiple testing correction (q < 0.05). Data from 28 squirrel monkeys are presented for 5557 probes. Abbreviations: dlPFC dorosolateral prefrontal cortex, vlPFC ventrolateral prefrontal cortex, vmPFC ventromedial prefrontal cortex. (XLSX 852 kb)

Additional file 3: Table S3. Sex differences. Genes targeted by probes with sex difference main effects (*P* < 0.05) that survived multiple testing correction (q < 0.05) across squirrel monkey prefrontal cortical regions. Data from 10 males and 18 females are presented for 82 probes. (XLSX 22 kb)

Additional file 4: Table S4. Correlations between qPCR and array. Determinations by qPCR correlated with array results regardless of whether prefrontal cortical regions were analyzed separately (18 females +10 males = 28 pairwise comparisons per region) or combined (3 regions x [18 females +10 males] = 84 pairwise comparisons). Abbreviations: dlPFC dorosolateral prefrontal cortex, vlPFC ventrolateral prefrontal cortex, vmPFC ventromedial prefrontal cortex. (XLSX 10 kb)
